# Cases of Rabies in Dogs

**Published:** 1814-03

**Authors:** 


### *For the Medical and Physical Journal.*

### *Cases of Rabies in Dogs; by* Mr. Youatt.

THE practitioners of the veterinary art owe much to the liberality of the teachers of human medicine. They are, fortunately, enabled, in some imperfect degree, to repay the obligation by the superior advantages which their profession gives them for the study of comparative anatomy and physiology.

In no respect can they contribute more essentially to the perfection of medical science, than by the light which their experience enables them to throw on the history of Rabies Canina; a disease yet involved in much obscurity, and the cure, or even alleviation of which, is a desideratum in the art.

To my partner, Mr. Blaine, the medical world is indebted for the only detailed and correct account of the symptoms of rabies in dogs, (with the exception, indeed, of Mr. Meynell, Medical Commentaries, vol. xix. p. 90) and from him a late writer on the bite of a rabid animal has unblushingly borrowed the most valuable part of his essay, though he had not the candor to acknowledge or even glance at his obligation.

The enclosed history of some cases of rabies canina, which occurred in my practice during the last year, is transmitted to you, as a very humble contribution to the knowledge of this dreadful malady. They are selected from many others, as clear and decisive in themselves; and each of them is marked by distinct and characteristic symptoms.

Case I.—January 18th, 1813.—Mr. G.'s terrier yesterday quarrelled with and bit another dog, with which he had been always friendly.

19th.—Having been costive two days, his master gave him some castor oil. In effecting this he was severely bitten. Alarmed at a ferocity which he had never before observed in the animal, Mr. G. sent for my partner, Mr. Blaine. The dog snapped at a stick, and shook it, but not without being a little teased. He howled frequently, with his head lifted up, but much of the bark was mixed with the howl. His head frequently drooped, and his eyes closed; he then suddenly started, lifted up his head, and howled.

20th.—To-day I saw him. He was busily employed in drawing the straw from his kennel, and shaking it violently. He instantly, and ferociously, flew at a stick. The howl was perfectly characteristic. He had lapped his urine, and had gnawed away his penis considerably below the glans. He yet knew his master's voice, and was obedient to his call.

call. He eagerly lapped milk and water, and readily swallowed it.*

At Mr. G.'s request he was immediately destroyed.

The trachea and œsophagus exhibited no morbid appearance. The stomach was very highly and generally inflamed, and completely filled with hair and straw. Several patches of inflammation were seen on the lungs. The diaphragm in some parts was livid. The duodenum and jejunum were violently inflamed, but the inflammation was lost in the ileum. The dog which was bitten by him died rabid five weeks after.

Case II.—March 3d.—Mr. L.'s terrier, very gentle and docile, had been two days costive and dull, and had obstinately refused all food. He now became suddenly furious, gnawed his kennel to pieces, and in the violence of his efforts broke all his incisor teeth. In the intervals of his violence his head drooped, and his eyes closed for a moment; he then suddenly sprang up, and recommenced his attack on the kennel.

4th.—His strength was much exhausted. His eyes had lost their ferocity, and had a dull greenish appearance. The sight of one eye was gone. The breathing laborious, with a peculiar choking noise. Eagerly seized his meat, carried it to his kennel, and laid on it. On giving him water in a shallow dish, he first lapped it eagerly, and then deliberately laid across it, burying his chest in it. Doubting whether this might not be accidental, he was rouzed, and the pan was removed to

---

* This fact completely refutes a very common opinion, that rabid animals are averse to water. A very similar one is related in the 20th volume of Duncan's Medical Commentaries, by Mr. Alexander Johnston. A dog supposed to be mad was dissected. Mr. J. remarks, "As we had always understood that a dog laboring under the hydrophobia would neither eat or drink, the stomach was inspected, and *to our great joy we found the stomach more than half full of food*, which had a most corrupt disagreeable smell, insomuch that we conjectured the animal had been eating human fæces. This circumstance proved an additional consolation to the mind of the poor man (who had been bitten), and led us to think the animal was not diseased." It may be right to add, that notwithstanding the wounded parts were entirely removed, and every precaution taken, the man fell a victim to the disease in five weeks from the time of the accident.—As the subject of Rabies possesses the strongest claims to our notice, both in regard to its fatality, the distressing and horrible character of its symptoms, and the obscurity in which it is still involved, we have great pleasure in announcing that we hope to publish, in our next Number, a valuable communication on the symptoms of rabies in dogs, by Mr. Blaine, whose attention to the diseases of animals is universally known.—Editors.

a little distance, yet within his reach: he immediately went and again laid across the water, immersing his chest in it. The water was then taken away, and soon after he eagerly scraped all his straw into a heap, and lay across it, with his stomach resting on it. He yet knew and fondled on his master. In the evening he died. He at no time gave the characteristic howl.

On opening the head, the longitudinal sinus and the vessels of the coats of the brain were beautifully turgid. The substance of the brain was reddish. No fluid in the ventricles. The pharynx and larynx were unaffected. The mucous membrane of the trachea was slightly, and the lungs were violently inflamed. Large patches, not spots, of inflammation appeared on the villous coat of the stomach. A considerable quantity of indigesta was contained in the stomach. The duodenum and jejunum were much inflamed, but the inflammation ceased in the ileum.

Case III.—August 7th.—A terrier, usually very gentle, was observed to be irritable and fretful; almost incessantly employed in hunting flies, except that for a moment his head suddenly drooped, and his eyes closed.

8th.—In the morning he seized his mistress's hand, and mumbled it, pretending to bite. He repeatedly did the same with her feet. The servant could scarcely drive him from *her* feet. He lapped eagerly, and took his food readily, but dropped it. In the afternoon he bit the gardener, and then fell furiously on the cat, and almost worried her to death. Had been costive three days.

9th.—To-day I first saw him. He was employed in tearing his bed and kennel to pieces, with strange diligence. Thirst excessive. Plunged his whole face in the water, yet could not swallow. His eyes were fierce. He flew furiously at a stick. His bark not changed. On my deciding that he was rabid, his master immediately shot him. The larynx, pharynx, trachea, and œsophagus were unaffected; but the left lung was slightly inflamed. The cardiac portion of the stomach was slightly inflamed, and distended with hair, leaves, straw, &c. The intestines were not affected.

Case IV.—October 4th.—Mrs. M.'s pug had for the last three days been unusually peevish. To-day he flew at his mistress, without provocation, though he had before been much attached to her. He then worried the cat, ran away, was seen to bite several dogs, and did not return until the next morning.

5th.—He was shut up in the wash-house, and meat and water were given him. He was not heard to bark or howl; but he gnawed the doors and windows to an extent that appeared almost incredible.

6th.—To-day I saw him. His meat was untouched, but the water all gone. He had lost all inclination to bite. I could not provoke him to bite. Staggered every moment, and towards evening was quite paralytic in his hind limbs. Purged violently. Knew his mistress, and was eager to be caressed. Died in the night. No howl.

The pharynx, larynx, œsophagus, trachea, and lungs unaffected. The stomach generally and highly inflamed, and quite empty. The intestines much inflamed, and considerably thickened through their whole extent.

Case V.—October 9th.—A terrier had for two days been unable to shut his mouth; had appeared eager to eat, but after many ineffectual attempts, dropped his meat. Imagining it to be a sore throat, the servants had given him several medicines, and two of them had scratched their hands against his teeth. The disorder apparently increasing, I was sent for. The lower jaw was completely paralytic. The tongue hung out, and was of a leaden color. The thirst extreme. Buried his nose in the water, but could not swallow. Flew furiously at me, but was unable to bite. A stick enraged him violently. Seemed to watch some imaginary objects in the air, and attempted to snap at them. Staggered as he walked. The head heavy, and eyes frequently closing. Knew the servants, and was obedient to them. Made no attempt to bite a dog which had been his companion. No howl.

Contrary to my request, he was immediately destroyed. The tongue was swollen, and of a leaden color at the tip. The stomach was empty. The fauces inflamed and swollen. The trachea and lungs unaffected. The vessels of its outer coat were very turgid. The villous coat was highly and generally inflamed, with spots of a deeper color. The bowels inflamed through their whole extent, and almost gangrenous in some parts.

Case VI.—October 17th.—Mrs. —— found a great quantity of blood in the bed of her white terrier, which evidently came from him, and was supposed to be vomited.

18th.—Very dull, and refused to eat; with much oppression of the head; was contented only when he could lay his head on his mistress's foot, a thing which he had never done before.

19th.—His lower jaw became slightly dependent. He now took some food, and after many ineffectual attempts, and frequently dropping it, swallowed it; lapped eagerly, plunging the whole of his face into the water; quarrelled with a cat, of which he had been very fond.

20th.—To-day I saw him. The lower jaw was paralyzed; the tongue hanging out, and of a leaden hue; the eyes glazed, glazed, and of the color of a green glass bottle. He staggered as he walked, and repeatedly fell. Watched some imaginary floating objects, and attempted to snap at them; the eye-lids frequently closing. The breathing was laborious, with a deep choaking noise. He was restless. He knew his mistress, fawned at her feet; attempted to lick my hand on patting him. No howl.

21st.—The deep choaking noise had gradually increased till noon, when he died. He had been unable to crawl from his bed since last evening, but was sensible to caresses until the last moment. Ten minutes before he died, he faintly wagged his tail when his mistress called to him.

The tongue was swollen and livid; the pharynx and larynx were inflamed; and the ramifications of the distended vessels of the trachea were very beautiful; the left lobe of the lungs highly inflamed, but the right lung scarcely affected; the whole of the villous coat of the stomach was highly inflamed, with the exception of a few spots which retained their natural color, and the pyloric orifice choked with straw; the parietes of the duodenum and jejunum were thickened; the mucous coat inflamed, but the inflammation gradually subsided, and was lost in the colon.

